# Recovery Through Deprescribing: A Case Study

**DOI:** 10.1192/bjo.2026.11889

**Published:** 2026-06-30

**Authors:** Amr Romeh, Laura Thorn, Linda Saji

**Affiliations:** Cardiff and Vale University Health Board, Cardiff, United Kingdom

## Abstract

**Aims:**

Memantine is an NMDA receptor antagonist used as pro-cognitive medication across the world. In the UK, it is licensed for moderate-to-severe stages of Alzheimer’s dementia. Memantine, like other medications, is associated with side effects that could be life-threatening in certain occasions. While clinicians mainly focus on medication initiation, titration and switch-over, deprescribing remains a critical step to uphold patients’ care especially in older adults and those who are more vulnerable to side effects.

**Methods:**

85-year-old lady with advanced mixed dementia on a psychiatric ward had a fall and a pelvic fracture. Following surgery, there was evident decline in her physical and mental health. She was nursed in bed all time and could not engage with post-operative rehabilitation. She became agitated on personal care interventions then started to present with impaired consciousness for extended periods during the day. The presentation continued to get worse to the extent that her impaired consciousness affected her diet and fluid intake and she lost significant amount of weight. Prolonged postoperative delirium was evident but there was no clear culprit to why her presentation is getting worse. Interestingly, her medication concordance was not affected for the majority of that period. It was felt that the patient could be slowly dying so we discussed the situation with palliative care team; they noticed some choreo-athetotic movements in the patient’s limbs which prompted reviewing her psychotropic medications which included Memantine 20mg. Once Memantine was discontinued, all the patient’s symptoms improved including her impaired conscious level and the abnormal limb movements.

**Results:**

Literature review highlighted rare associations between Memantine, such abnormal movements and impaired consciousness. This patient’s recovery from this post-operative state was lengthy and was eventually achieved after addressing “a blind spot” which was the prescribed medication Memantine. The main reason to stop Memantine was the patient’s abnormal movements but it was also responsible for the impaired conscious level. Once Memantine was discontinued, the patient’s mental and physical states changed dramatically. She became more responsive and her conscious level was back to normal. The choreo-athetotic movements were no longer visible as well.

**Conclusion:**

Deprescribing is a valuable yet missed tool for improving patient outcomes. It is commonly undertaken when a medication is clearly linked to adverse effects and is often paired with prescribing alternatives. However, deprescribing is frequently overlooked when symptoms are not evidently medication-related. Implementing a proactive deprescribing process can help reduce iatrogenic harm and improve overall clinical outcomes.

